# Research Progress on Multi-Component Alloying and Heat Treatment of High Strength and Toughness Al–Si–Cu–Mg Cast Aluminum Alloys

**DOI:** 10.3390/ma16031065

**Published:** 2023-01-25

**Authors:** Mingshan Zhang, Yaqiang Tian, Xiaoping Zheng, Yuan Zhang, Liansheng Chen, Junsheng Wang

**Affiliations:** 1 Key Laboratory of the Ministry of Education for Modern Metallurgy Technology, North China University of Science and Technology, Tangshan 063210, China; 2 School of Materials Science and Engineering, Beijing Institute of Technology, Beijing 100081, China; 3 Advanced Research Institute of Multidisciplinary Science, Beijing Institute of Technology, Beijing 100081, China

**Keywords:** Al–Si–Cu–Mg cast aluminum alloys, multi-component alloying, heat treatment, microstructural regulation, mechanical property

## Abstract

Al–Si–Cu–Mg cast aluminum alloys have important applications in automobile lightweight due to their advantages such as high strength-to-weight ratio, good heat resistance and excellent casting performance. With the increasing demand for strength and toughness of automotive parts, the development of high strength and toughness Al–Si–Cu–Mg cast aluminum alloys is one of the effective measures to promote the application of cast aluminum alloys in the automotive industry. In this paper, the research progress of improving the strength and toughness of Al–Si–Cu–Mg cast aluminum alloys was described from the aspects of multi-component alloying and heat treatment based on the strengthening mechanism of Al–Si–Cu–Mg cast aluminum alloys. Finally, the development prospects of automotive lightweight Al–Si–Cu–Mg cast aluminum alloys is presented.

## 1. Introduction

With the increasingly serious problems of environmental pollution and energy crisis, the automotive industry is developing in an environmentally friendly direction. Relevant data shows that for every 10% weight reduction of automobiles, fuel consumption is reduced by 8%, and exhaust emissions are reduced by 4% [1]. Therefore, automobile lightweighting is the most economical way to reduce energy consumption and mitigate environmental damage [2,3]. In recent years, automotive lightweight materials have been fiercely competitive, and aluminum alloys have been the first choice for automotive lightweight materials due to a series of advantages such as low cost, high specific strength, and good corrosion resistance [4,5].

Among automotive aluminum alloys, cast aluminum alloys are widely used in automotive parts due to their good casting moldability and economy [6]. Cast aluminum alloys usually include Al–Si, Al–Mg, Al–Cu, and Al–Zn alloys. Al–Si cast aluminum alloys have the advantages of good fluidity, wear resistance, high mechanical strength, and high yield, therefore, they are widely used in automobile components. Moreover, Al–Si–Cu–Mg cast aluminum alloys are widely used in cylinder heads and engine blocks due to their high strength-to-weight ratio, good thermal stability and castability [7,8]. At the same time, with the increase of the demand for product performance in the automotive industry, the performance of Al–Si–Cu–Mg cast aluminum alloys have been put forward higher requirements for automotive parts. The development of high-strength Al–Si–Cu–Mg cast aluminum alloy to meet the room temperature and high temperature performance requirements of automotive parts can further promote the application of cast aluminum alloys in automobiles.

In order to realize the development of high-strength Al–Si–Cu–Mg cast aluminum alloy, researchers have been committed to the research of alloy composition, microstructure and mechanical properties. Studies show that the mechanical properties and fracture behavior of cast aluminum alloy mainly depend on the microstructure characteristics of different scales, and alloy elements and heat treatment play an important role in the microstructure evolution and mechanical properties regulation [9,10]. Cao et al. [11] found that the addition of the microalloying element Cd not only promoted the precipitation of θ’-Al_2_Cu phase, but also refined the size of θ’-Al_2_Cu phase. Zhang et al. [12] found that the Sc element can refine grain and change the morphology of eutectic Si, and form a new nano-precipitated phase to provide an additional fine grain strengthening and precipitation strengthening effect. Wang et al. [13] obtained a mixture of β″-Mg_5_Si_6_, θ’-Al_2_Cu and Q’-Al_5_Cu_2_Mg_8_Si_6_ nanoprecipitated phases by adjusting artificial aging parameters, resulting in excellent mechanical properties of Al–Si–Cu–Mg alloy. Fang et al. [14] optimized the two-stage solution treatments of Al–Si–Cu–Mg alloy to obtain a high and uniform solute concentration without overburning of the Cu-containing phase, thus improving the solution strengthening. However, although the research on multi-component alloying and heat treatment of Al–Si–Cu–Mg cast alloy has been extensively studied, there is still little comprehensive and exhaustive research on it based on the strengthening mechanism of casting alloy, which is not conducive to the development and application of high strength and toughness Al–Si–Cu–Mg cast aluminum alloys.

Therefore, this article mainly introduces the microstructure regulation of Al–Si–Cu–Mg cast aluminum alloys by the optimization of multi-component alloying and heat treatment, and describes the research progress in improving its strength and toughness based on the strengthening mechanism of Al–Si–Cu–Mg cast aluminum alloys. It provides theoretical guidance for high strength and toughness Al–Si–Cu–Mg cast aluminum alloys for automobile.

## 2. Strengthening Mechanism

The strength of the alloy materials is related to the ability to resist the deformation of the material. However, the deformation of the alloy materials is mainly related to the movement of the dislocation. Therefore, the methods that can improve the resistance to the movement of the dislocation can increase the strength of the material. At present, the main strengthening mechanisms for cast aluminum alloys include solid solution strengthening, fine grain strengthening, and second phase strengthening.

### 2.1. Solid Solution Strengthening

Solid solution strengthening refers to the alloy elements dissolve in the aluminum matrix, causing the alloy element to elastically and electrochemically interact with the dislocation, forming lattice distortions, thereby hindering the movement of the dislocation and improving the strength of the alloy material. The strength of the alloy is mainly related to the concentration of solute atoms in the matrix and the radius difference with the matrix atoms. The yield strength and solute atom concentration of the alloy can be expressed by the theoretical Equation (1): the greater the number of solute atoms, the more obvious the strengthening effect of the alloy. In addition, the larger the radius difference between the solute atoms and the matrix atoms, the greater the degree of lattice distortion caused, and the better the solid solution strengthening effect.

(1) $$\sigma =\sigma_{0}+kC^{m}$$

where: σ—Yield strength;

$\sigma_{0}$—Yield strength of pure metal;

C—Solute atomic concentration;

k,m—Constants related to solute atom type.

### 2.2. Fine Grain Strengthening

Fine grain strengthening is one of the important strengthening mechanisms of cast aluminum alloys. The smaller the grain size of the alloy material, the more the grain boundaries, which effectively hinder the movement of dislocations and improve the strength of the alloy. The relationship between the fine grain strengthening and the yield strength of the alloy material conforms to the Hall–Petch theoretical formula, As shown in Equation (2) [15]. It can be known from the formula that the yield strength of alloy materials increases with decreasing grain size at room temperature. The refinement of the cast aluminum alloy structure is mainly the refinement of α-Al dendrites. By refining the dendrites, the dendrite arm spacing is reduced and the alloy strength is improved.

(2) $$\sigma_{s}=\sigma_{i}+k\cdot d^{-1/2}$$

where: $\sigma_{s}$—Yield strength;

$\sigma_{i}$—material constant for the starting stress for dislocation movement;

*k*—Constant;

d—Average grain diameter.

### 2.3. Second Phase Strengthening

**Figure 1:**
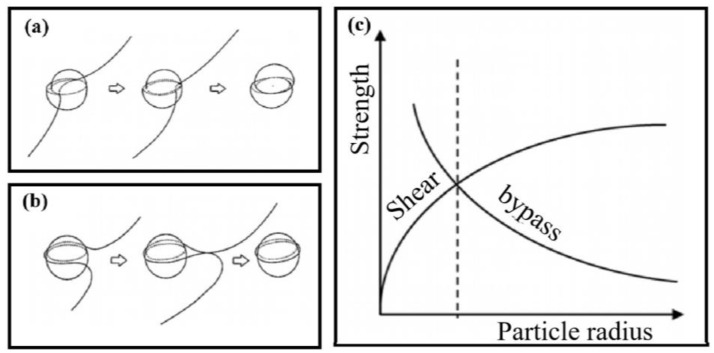
Relationship between dislocation motion and precipitation phase [17]: (**a**) Friedel mechanism. (**b**) Orowan mechanism. (**c**) Relationship between precipitation phase size, alloy strength, and dislocation motion.

The strengthening effect of the second phase is derived from the ability of the second phase to hinder the motion of dislocations. The strength is determined by the size and distribution of the precipitates and the coherence of the precipitates with the matrix [16]. The interaction between dislocations and the second phase can be described by shear and bypass mechanisms. Figure 1 is the relationship between dislocations and precipitated phases [17,18,19]. The small and not too hard precipitated phases are sheared when they encounter dislocation movement (the shear mechanism is also called the Friedel mechanism), as shown in Figure 1a. When the size of the precipitate is larger and harder, the dislocation bypasses the precipitate by bending (bypasses mechanism), as shown in Figure 1b. As long as the precipitates are sheared by dislocations, the strength of the alloy will increase. With the further growth of the precipitated phase, the shearing process becomes quite difficult. Dislocations are more conducive to bypassing the precipitates, resulting in the reduction of alloy strength. As shown in Figure 1c, the highest strength is obtained when the dislocations pass through the precipitate with shear and bypassing with equal probability. Generally, the bypass mechanism includes two mechanisms, one is Orowan shear loops, and the other is Hirsch prismatic loops [20]. Dislocation with any character angle will form dislocation shear loops around the precipitate after bypassing the precipitate, resulting in Orowan strain hardening. If all dislocations are edge-oriented, when bypassing the precipitate, a prismatic loop will form on either side of the precipitate when bypassed, which is called Hirsch looping. In addition, it has been shown that two dislocations can form a dislocation dipole, which plays an important role in the plastic deformation of materials, especially fatigue and creep [21].

The strengthening effect of different strengthening mechanisms depends on different factors, solid solution strengthening mainly depends on the supersaturation of solid solution atoms in aluminum matrix, fine grain strengthening mainly depends on the degree of grain and phase refinement, and the second phase strengthening mainly depends on the type, size and distribution of the second phase [22]. According to the strengthening mechanism principle and influencing factors of cast aluminum alloy, the methods of multi-component alloying and heat treatment process optimization are usually adopted to improve the microstructure and distribution of cast aluminum alloy and improve the strength of cast aluminum alloy.

## 3. Multi-Component Alloying Optimization

The alloy composition of cast aluminum alloy determines the microstructure of the alloy, and multicomponent alloying is one of the main methods to improve the properties of cast aluminum alloy. Multi-element alloying usually involves the optimization of main alloying elements and trace elements.

### 3.1. Main Alloying Elements

**Figure 2:**
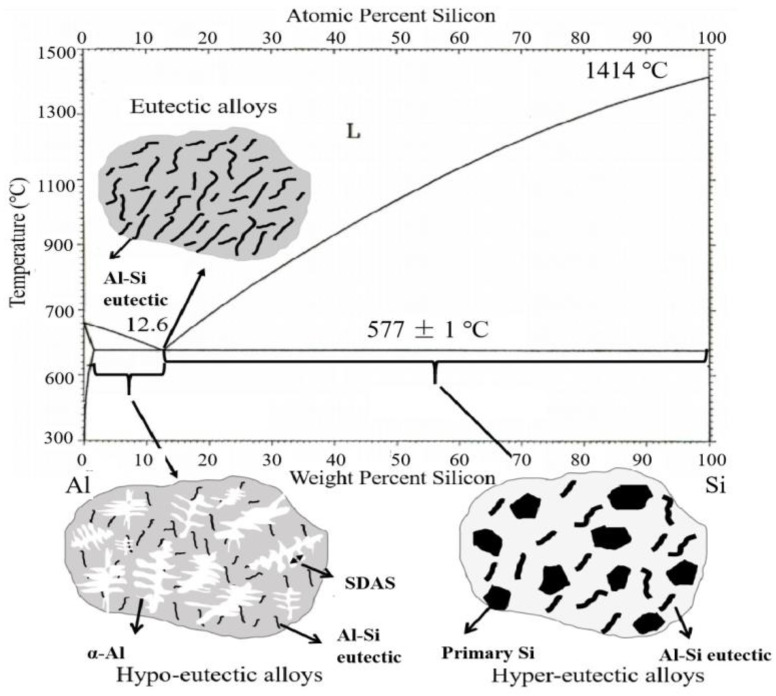
Binary phase diagram and as-cast microstructure diagram of Al–Si cast aluminum alloy.

The solid solubility of Si in aluminum is 1.65 wt.% (577 °C), but decreased to 0.05 wt.% at room temperature [23]. The addition of Si to aluminum alloy can reduce the volume shrinkage and improve the fluidity, thus improving the casting performance of aluminum alloy [24]. For Al–Si cast aluminum alloy, the Si content is usually selected between 5% and 23%, and the Al–Si eutectic reaction occurs at 12.6 wt.% Si [25]. As shown in Figure 2, different Si contents lead to different microstructure of Al-Si alloys. The influence of Si elements on the mechanical properties of the alloy is related to the Si content and the morphology of eutectic silicon particles: when the Si content is between 4 and 20%, the yield strength first increases and then decreases with the increase of Si content [26]. In addition, the addition of Si to the alloy often results in the formation of large plate-strip eutectic structure, and the morphology of eutectic Si is usually changed from large plate-strip to small fibrous morphology through modification to improve the toughness of the alloy [27].

Mg is one of the important solid solution strengthening elements in cast aluminum alloy. Studies have shown that Mg has a large solid solubility in aluminum, which can reach up to 17.4 wt.% at 450 °C, while only 0.3~0.7 wt.% in Al–Si cast aluminum alloys at room temperature. Therefore, Mg_2_Si can be formed when Mg and Si elements are added together [28]. In addition, after the aging heat treatment, the dispersion strengthening phase of the alloy β″-Mg_5_Si_6_ can be precipitated to improve the alloy strength [29,30].

Cu is also one of the important solid solution strengthening elements of cast aluminum alloy. Since the maximum solid solubility of Cu in aluminum is 5.65 wt.% (546 °C), but it is very small at room temperature, the alloy will precipitate intermetallic compounds during the solidification process, such as θ-Al_2_Cu, Q-Al_5_Cu_2_Mg_8_Si_6_ and so on. The dispersion-strengthened phases θ’-Al_2_Cu and Q’-Al_5_Cu_2_Mg_8_Si_6_ are also precipitated after aging heat treatment, which improve the age hardening strength of the alloy [31,32]. In addition, because the θ ‘and Q’ metastable phases have strong resistance to roughening at high temperatures, the addition of Cu will improve the high-temperature thermal stability of Al–Si cast aluminum alloys [33].

### 3.2. Microalloying

At present, the research on the main alloying elements and their proportions of cast aluminum alloys is very mature. Microalloying is an ideal choice to further improve the strength and comprehensive performance of the alloy. The study of microalloying elements in Al–Si–Cu–Mg cast aluminum alloys usually involves the following aspects [34]: (1) modification; (2) grain refinement; (3) formation of new precipitation strengthening phase.

#### 3.2.1. Modification

The eutectic Si of Al–Si–Cu–Mg cast aluminum alloys usually grows in the form of coarse flakes or needles under natural conditions. The primary Si is often polygonal and needle-like. These silicon particles are the source of stress concentration, and the size and morphology of silicon particles have an important impact on the mechanical properties of the alloy, especially the toughness of the alloy [22]. Therefore, silicon particle usually is modified during the solidification process to transform coarse flaky or needle-like eutectic Si into a fine fiber-like morphology and refine the size of the primary Si, thereby improving the strength and toughness of the alloy [35].

The modification methods of eutectic Si include chemical modification and quenching modification. The chemical modification method is widely used in production due to its simple operation, obvious modification effect and high economic benefit. Generally, modifiers such as Sr, Na, Sb, Ba, Ca, Y, Ce, and Eu are selected to modify the eutectic Si. Sr is widely used in hypoeutectic and eutectic Al–Si cast aluminum alloys due to its advantages of good reproducibility, long-term effective modification time, no corrosion to equipment, insensitivity to cooling rate and low environmental pollution [36]. The method of adding P to the melt is usually used for the modification of the primary Si particles. P and Al react with each other to form an AlP compound [37]. In addition, the composite modification can achieve multiple modification effects, which can simultaneously modify eutectic Si and primary Si particles, but there are also some modification elements that inhibit or poison each other, such as Sr-Na, P-Ca and P Eu, etc., which need to be avoided when preparing Al–Si cast aluminum alloys.

**Figure 3:**
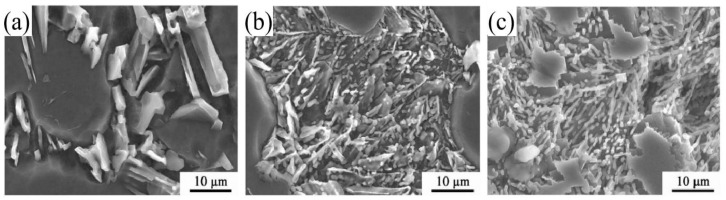
Typical morphology of eutectic Si in Al-Si-Mg cast aluminum alloy with different Sc contents: (**a**) 0 wt.% Sc; (**b**) 0.2 wt.% Sc; (**c**) 0.8 wt.% Sc [38].

Rare earth elements have modification effect on eutectic Si, among which Sc has a greater modification effect. Xu et al. [38] studied the modification effect of Sc element on eutectic Si. As shown in Figure 3, with the increasing content of Sc element, the morphology of coarse flake eutectic Si transforms into finer fibrous morphology. The addition of 0.8 wt.% Sc resulted in a decrease in the aspect ratio of eutectic Si from 27 to 2. In addition, studies have shown that Mg also has modification effect on eutectic Si morphology by inhibiting the growth of eutectic Si [39]. Samuel et al. [40] found that when the Mg content was lower than 0.35 wt.%, the modification effect of Mg element on eutectic Si was not obvious, but when the Mg content was increased to 0.6 wt.%, it had a modification effect on eutectic Si.

**Figure 4:**
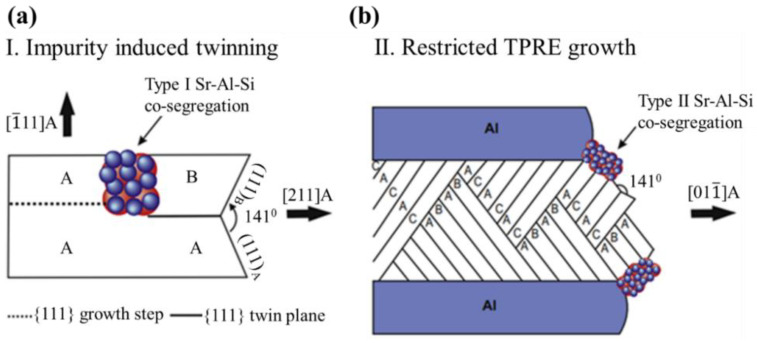
Schematic representation of (011) plane projection of eutectic Si [43]: (**a**) Type I rod-like Sr-Al-Si co-segregation which promotes twinning by changing the stacking sequence; (**b**) Locations of type II extended rod-like Sr–Al–Si co-segregations within the eutectic Si at the re-entrant edges or growing surfaces, which inhibits the growth of eutectic Si.

Many assumptions about the research on the modification mechanism of eutectic Si are based on the theoretical exploration that the modified atoms inhibit the nucleation and growth of eutectic Si. In recent years, the widely accepted modification mechanisms of eutectic Si are mainly the poisoning of twin plane re-entrant edge (TPRE) proposed by Hamilton and Seidensticker in the 1960s and the impurity-induced twinning (IIT) mechanism proposed by Lu and Hellawell [41,42]. Timpel et al. [43] explored the effect of Sr containing particles with different morphologies on the modification of eutectic Si, and found that the Sr containing particles with different morphologies and sizes have different mechanisms for the modification of eutectic Si. As shown in Figure 4a, nanocrystalline rod-like Sr particles lead to the formation of multiple twins in Si, which grow in different crystal growth directions (IIT); Figure 4b shows the growth of eutectic Si is restricted by the Sr-containing nanocrystalline strips (TPRE).

**Figure 5:**
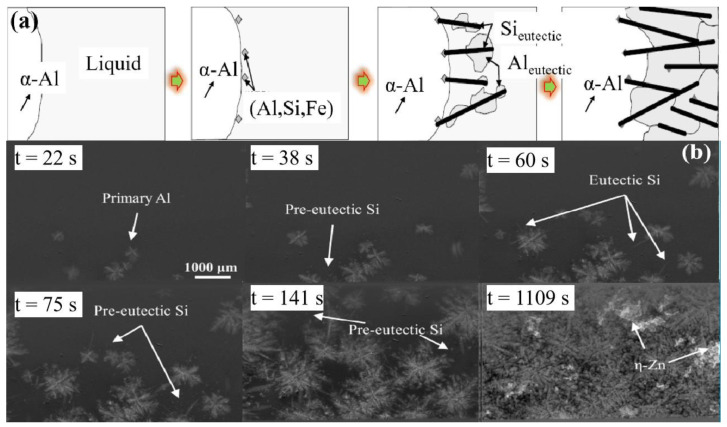
Nucleation and growth of eutectic Si: (**a**) Mechanism diagram of nucleation and growth of eutectic Si [47]; (**b**) in situ observation of eutectic Si nucleation and growth process in Al-40Zn-5Si alloy [50].

Most studies on the modification mechanism of eutectic Si is based on the nucleation and growth of eutectic Si [44,45,46]. Figure 5a shows the mechanism of eutectic Si nucleation and growth of hypo-eutectic Al–Si alloy [47]. Figure 5a shows that when the eutectic temperature is reached, the eutectic Si nucleates on the second phase β-(Al, Si, Fe), and then grows into the liquid in a thin morphology. Most studies on the mechanism of eutectic Si nucleation and growth usually investigate by means of cooling curve analysis, discontinuous solidification experiment and the cellular automata (CA) model [48,49]. However, these methods cannot capture the solidification morphology accurately in real time, and the simulation results are different from the experimental results. The synchronous X-ray method can realize the in-situ observation of the solidification process, which provides a good way for the real-time observation of eutectic Si. Mao et al. [50] studied the nucleation and growth process of eutectic Si in Al–Si alloy using synchronous X-ray technology. Due to the similar atomic number and density of Al and Si, it is difficult to distinguish the two morphologies by contrast. In order to improve the contrast at the solid–liquid interface and further distinguish the morphology of eutectic Si more easily, the researchers added Zn element to the Al–Si alloy. As shown in Figure 5b, the nucleation and growth process of eutectic Si can be clearly observed by in-situ X-ray observation.

#### 3.2.2. Grain Refinement

The refinement structure of Al–Si cast aluminum alloys is mainly to promote the formation of primary α-Al dendrites into fine equiaxed grains. The formation of equiaxed grains can also prevent the occurrence of porosity and shrinkage, and also reduce the tendency of hot cracking and improve the overall mechanical properties of the alloy [51]. Among many methods of grain refinement, the most effective and economical method of adding grain refiner is usually selected in industrial production. The selection of refiners has undergone a series of developments: from the early salt refiners (K2TiF6, KBF4, etc.) to the intermediate alloys (Al–Ti, Al–B, Al–Ti–B, Al–BC and Al–Ti–C, etc.). The addition of intermediate alloys has also evolved from the early bulk or ingot to rod-shaped intermediate alloys [52].

As the solid solubility of trace elements Zr, Ti, V, Er and Sc in Al solid solution gradually decreases with the decrease of temperature, Al_3_M (M is Zr, Ti V, Er or Sc etc.) phase is formed during the solidification of the alloy. Due to the small mismatch between Al_3_M particles and α-Al solid solution, and causing composition undercooling, it can be used as an effective heterogeneous nucleation point for α-Al during solidification, thereby refining the structure [53,54]. Liu et al. [55] found that the addition of 0.14 wt.% Zr to the Al–Si–Cu–Mg cast aluminum alloy has a significant refinement effect on the grain size of the alloy. The grain size of the base alloy is 335 μm. while the grain size was reduced to 253 μm after the addition of 0.14 wt.% Zr. Studies have shown that the addition of trace elements Zr, Ti, V, Er, and Sc to the melt will cause the composition undercooling [56]. The mechanism of solute elements on the grain refinement can be explained by the growth restriction factor (GRF) relationship, as shown in Equation (3). GRF refers to the degree of constraint of solute atoms on the solid–liquid interface growth when new grains grow into the melt. Therefore, the above formula indicates that GRF increases with the increase of trace element concentration, and the degree of constraint on grain growth increases, resulting in more obvious refinement effect.

(3) $$GRF=\sum {mc}_{0}\;(k-1)$$

where: *m*—Liquidus slope;

$c_{0}$—Solute atomic concentration;

k—Solute distribution coefficient during solidification.

#### 3.2.3. New Precipitated Strengthened Phase

In addition to forming Al_3_M phase during solidification, trace elements such as Zr, Ti, V, Er and Sc can also precipitate Al_3_M phase during aging heat treatment. Al_3_M phase is dispersed and fine distributed in the aluminum alloy matrix, which plays a role of pinning on dislocation, impeding dislocation movement and effectively improving alloy strength [57]. It is found that the tensile and low-cycle fatigue properties of the alloy under peak aging conditions are significantly improved due to the generation of dispersing fine Al_3_M phase, and the yield strength was increased by 60–87% when Ti, Zr and V were added to Al–Si–Cu–Mg cast aluminum alloys [58]. In addition, the thermal stability of Al_3_M phases are good due to the low solubility and weak diffusion capacity of these elements in aluminum matrix, which can improve the high-temperature mechanical properties of the alloy [59]. Shaha et al. [60] studied the influence of Zr, Ti and V elements on the high-temperature mechanical properties of Al–Si–Cu–Mg cast aluminum alloys, and found that under the peak aging condition, the yield strength and ultimate tensile strength of the alloy with trace elements increased by 30% and 5% compared with that of the alloy without addition due to the effect of the dispersion and precipitation phase.

**Figure 6:**
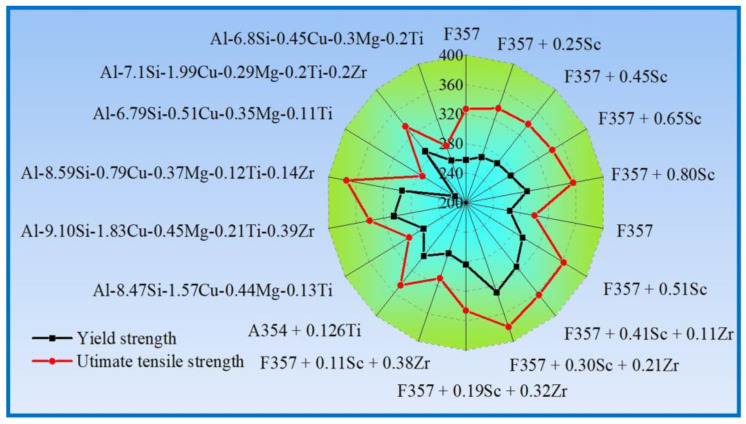
Investigation of the strength of Al–Si alloy. This figure is reproduced based on [38,55,61,62,63,64].

Xu et al. [38] studied the influence of different Sc content on the microstructure and mechanical properties of the F357 cast aluminum alloy, and found that with the increase of Sc content, when Sc increased to 0.8 wt.%, the eutectic silicon morphology and secondary dendrite arm spacing of the F357 alloy were significantly refined. In addition, the yield strength and ultimate tensile strength of the alloy increased from 258 MPa and 327 MPa to 289 MPa and 356 MPa, respectively, and the elongation increased from 6.9% to 12.8% due to the formation of fine Al_3_Sc strengthening phase under T6 peak aging heat treatment. Rahimian et al. [61] studied the effect of Zr on the microstructure and properties of Al–Si–Cu–Mg alloy, and found that after T6 peak aging treatment, Al–Si–Zr–Ti precipitates with a fine dispersion and a size of 80~200 nm were formed, which together with θ’ and Q’ phases formed the precipitation strengthening phase of the alloy. The yield strength and ultimate tensile strength of the alloy increased from 261 MPa and 282 MPa to 291 MPa and 335 MPa, respectively. Researchers have conducted a lot of research on the influence of trace elements Zr, Ti, V and Sc on the mechanical properties of Al–Si cast aluminum alloy, and it has been shown that the addition of microalloying elements is very beneficial to improve the mechanical properties of Al–Si cast aluminum alloy [38,55,61,62,63,64]. Figure 6 shows the strength of microalloyed Al–Si cast aluminum alloy under peak aging in recent years. The results show that the yield strength and ultimate tensile strength of Al–Si cast aluminum alloy are approximately 250~330 MPa and 270~380 MPa, and the yield ratio is between 0.79–0.93 after microalloying regulation. The yield ratio of some alloys is still very high, resulting in low formability and use safety at room temperature.

## 4. Heat Treatment Process Optimization

The heat treatment of Al–Si cast aluminum alloys usually includes solution treatment and aging treatment (natural aging at room temperature or artificial aging at high temperature). Heat treatment to improve the strength of cast aluminum alloy is mainly due to the solid solution strengthening, as well as aging precipitation strengthening effect. However, the heat treatment process has a great impact on the solid solution effect and precipitation phase of the alloy. Therefore, the optimization of heat treatment process is very important for improving the alloy structure and improving the strength and toughness of casting parts [65,66,67].

### 4.1. Solution Treatment

Solution treatment refers to holding the alloy for a certain time after heating to a specific temperature, and then quenching to room temperature to obtain super-saturated solid solution, which provides a greater driving force for late aging precipitation [68,69,70]. Generally, the solution temperature should be 5–10 °C lower than the eutectic temperature of eutectic phase with low melting point to prevent over-burning [49]. Solution treatment can dissolve intermetallic compounds (such as Mg_2_Si phase, Al_2_Cu phase and Al_5_Cu_2_Mg_8_Si_6_ phase) produced during solidification, and can also change the morphology of Si particles. The dissolution of Si particles in the solid solution process roughly undergoes three processes: crushing, spheroidizing and coarsening [71]. However, the dissolution and homogenization of intermetallic compounds of Al–Si–Cu–Mg cast aluminum alloys are relatively complicated due to the variety of primary phases. The solid solution process of intermetallic compounds is also the process of uniform diffusion of alloying elements. The short solution time does not dissolve all intermetallic compounds, and the longer solution time leads to the coarsening of the microstructure and the generation of secondary holes, as well as unnecessary energy consumption [72,73].

#### 4.1.1. Dissolution Characteristics of Intermetallic Compounds

**Figure 7:**
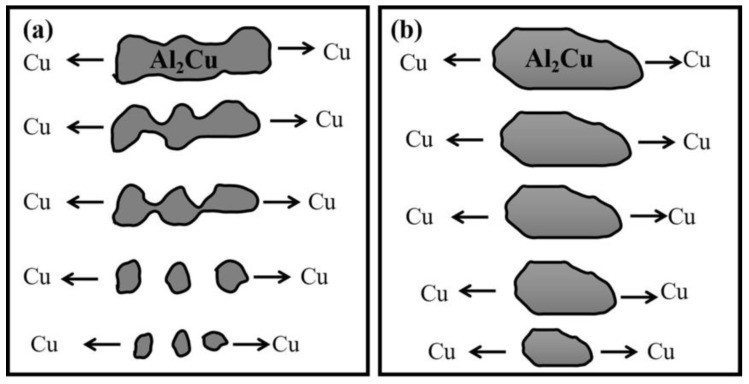
Dissolution process of Al_2_Cu particles with different morphologies [17]: (**a**) eutectic Al_2_Cu; (**b**) blocky Al_2_Cu.

The Al–Si–Cu–Mg cast aluminum alloys usually dissolve the Mg_2_Si phase, Al_2_Cu phase and Al_5_Cu_2_Mg_8_Si_6_ phase during the solid solution process. The time required for solid solution depends on the dendrite arm spacing, the primary phase and solution treatment temperature, etc. [74,75]. The dissolution process of Mg_2_Si phase is very rapid due to the large diffusion rate of Mg element in Al. Rometsch [76] studied the solution process of A356 and A357 alloys at 540 °C and found that the A356 alloy could complete the solution and homogenization process of Mg_2_Si phase within 15 min due to the low Mg content (0.40 wt.%) and the small secondary dendrite arm spacing (SDAS) (40 m). Even for A357 (0.62 wt.% Mg) with a large SDAS (55 m), the solution and homogenization time of Mg_2_Si phase only needed 50 min. The dissolution of Al_2_Cu phase is more difficult than that of Mg_2_Si phase due to the low diffusion rate of Cu in Al. Moreover, the Al_2_Cu phase in the as-cast structure has a variety of morphologies: for example, the blocky Al_2_Cu phase, eutectic Al_2_Cu phase or two kinds of mixture, and the solubility of Al_2_Cu phase with different morphologies is also different [77,78,79]. The dissolution process of Al_2_Cu phase with different morphologies in the solid solution process is shown in Figure 7 [17]: the eutectic Al_2_Cu phase dissolves into smaller particles through fragmentation, and then spheroidizes and finally dissolves through the diffusion of Cu to the surrounding matrix. However, the blocky Al_2_Cu phase was gradually dissolved by spherification and diffusion which takes a longer time. Samuel [80] studied the dissolution characteristics of the eutectic and bulk Al_2_Cu phases. The study found that even if the solution treatment is performed in the range of over-burning temperature (540 °C), the bulk Al_2_Cu phase still exists after solid solution for 24 h, and the eutectic Al_2_Cu phase has completely dissolved. In addition to the formation of Mg_2_Si phase and Al_2_Cu phase, Q-Al_5_Cu_2_Mg_8_Si_6_ phase is also formed during the solidification. The Q phase is distributed independently of script morphology, or coexists with Al_2_Cu and eutectic Si in the final solidification stage to form eutectic morphology [81]. The Q phase may dissolve or not dissolve or even precipitate during the solid solution process, and its dissolution characteristics are related to alloy composition [82]. Lasa et al. [83] investigated the Q phase solution process in Al–Si–Cu–Mg cast aluminum alloys with different Cu contents: for high Cu (4.4 wt.% Cu), different Mg contents (0.58–1.30 wt.% Mg) alloy, the content of Q phase in the solution process is almost unchanged; However, for alloys with low Cu (1.37 wt.% Cu) and high Mg (1.30 wt.% Mg), the quantity of Q phases increased after the solution at 500 °C for 5 h. This is mainly due to the higher Mg content causing more Q phases to be formed in the equilibrium state, but the number of actual Q phases is lower than the equilibrium state due to the faster solidification rate in the actual solidification process. However, the solid solution process is slower than the solidification process, and close to the equilibrium state condition, which leads to the re-precipitation of the unprecipitated Q phase during the solidification process. In addition, the dissolution characteristics of phase Q are also related to the solution temperature. Colley et al. [84] studied the dissolution characteristics of Q phase of Al—8.3 wt.% Si-2.8 wt.% Cu—0.5 wt.% Mg alloy at different solution temperatures and found that Q phase did not dissolve after 24 h at 480 °C, while almost all dissolved after 24 h at 505 °C.

#### 4.1.2. Application of Two-Step Solution and Thermodynamic Calculation in Solution Heat Treatment

In order to avoid over-burning of Cu-containing phase, the solution temperature of Al–Si–Cu–Mg cast aluminum alloys should not exceed the eutectic temperature at the low melting point, usually around 490–505 °C. However, there will still exist the undissolved flake Al_5_Cu_2_Mg_8_Si_6_ phase and the blocky Al_2_Cu phase after the solid solution treatment. In order to overcome this problem, Al–Si–Cu–Mg cast aluminum alloys is usually treated by two-step solution treatment. Through two-step solution treatment, Cu-containing intermetallic compounds can be completely or mostly dissolved, and the degree of homogenization of alloying elements is also improved, thus improving the strength and toughness of the alloy. Sokolowsk’s [85] research found that the Cu-containing phase is significantly refined and the number significantly reduced by the two-step solution treatment (8 h/495 °C + 2 h/520 °C) compared to the traditional single-step (8 h/495 °C) solution treatment, and the tensile strength of the aged alloy increased from 200 MPa to 240 MPa, and the elongation increased from 0.6% to 1.6% compared with the single-step solution treatment after aging at 250 °C for 5 h.

The aluminum alloy industry has made some progress in equipment and processing, but there are still some deficiencies in basic theoretical research such as heat treatment process design. Most of the research on heat treatment process design still focuses on trial and error. With the introduction of integrated computational materials engineering (ICME) in 2008, the traditional research model of “experience optimization”, characterized by a large amount of experience accumulation and simple cyclic trial and error, is broken through, and multi-scale simulation and experimental tools have been gradually combined with databases to integrate design and manufacturing. With the continuous development of thermodynamics and phase diagram calculation, the phase diagram calculation method (CALPHAD) is based on the laws of thermodynamics to calculate the phase diagram, diffusion and other related information of multi-component alloys by means of energy minimization [86]. The CALPHAD can effectively predict the phase types and temperatures under alloy equilibrium conditions by combining computer software and thermodynamic database, thus providing theoretical guidance for the design of heat treatment process [87]. In addition, Scheil–Gulliver model is used to predict the solidification path of the alloy to predict the phase formation during solidification [88].

**Figure 8:**
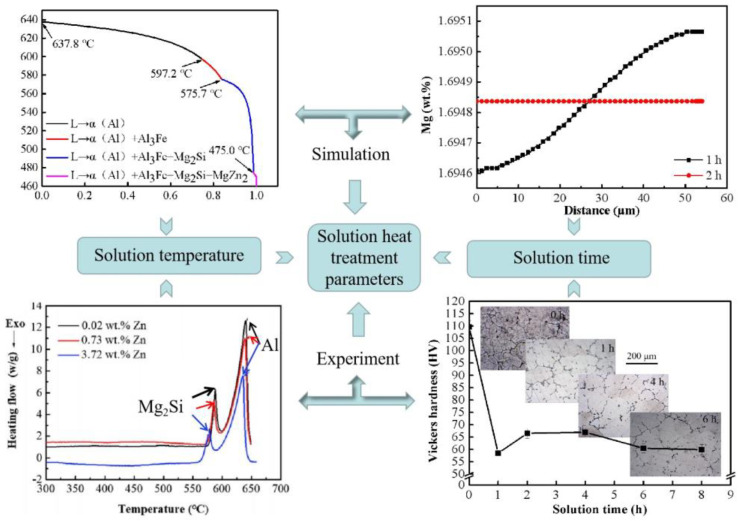
Optimization of solution heat treatment parameters by combining thermodynamic calculation and experiment [90].

CALPHAD is one of the important methods in integrated computational simulation. Many researchers use this method to solve the problems in phase transition and heat treatment process optimization. Li et al. [89] used Pandat software to study the equilibrium phase diagram of Al–Zn–Mg–Si alloy, and found that α-Al, Si, Mg_2_Si and MgZn_2_ phases existed at 573 K, which was well verified by scanning electron microscopy (SEM) and differential scanning calorimetry (DSC), providing theoretical guidance for phase prediction. Zhang et al. [90] investigated the effects of Zn addition and related heat treatment parameters on the mechanical properties of Al–Si–Mg alloys by using a combination of experimental and thermodynamic methods. In addition, the CALPHAD method was used to optimize the heat treatment process parameters. As shown in Figure 8, the non-equilibrium solidification path was predicted by thermodynamic calculation, and the temperature of low melting point eutectic phase was measured by DSC experiment. In order to prevent the eutectic phase from over-burning, the solution temperature should be lower than the low melting point eutectic phase temperature. On the other hand, the homogenization and diffusion of elements is also the purpose of solution heat treatment. As shown in Figure 8, thermodynamic software was used to predict the time for elements to reach diffusion homogenization at a specific solution temperature, and the best solution time was obtained by combining the evolution of microstructure and hardness at different solution times.

### 4.2. Aging Treatment

Aging treatment is one of the important means to improve the performance of Al–Si–Cu–Mg cast aluminum alloys. Aging means that supersaturated solid solution precipitates finely dispersed solute clusters or precipitated phases from the matrix at room temperature (natural aging) or at high temperature (artificial aging). These solute clusters/precipitated phases can effectively hinder the movement of dislocations, thereby achieving the precipitation strengthening [91,92].

Al–Si–Cu–Mg cast aluminum alloys precipitate strengthening phase (β″-Mg_5_Si_6_, θ’-Al_2_Cu and Q’-Al_5_Cu_2_Mg_8_Si_6_, etc.) to improve the aging hardening effect after aging heat treatment [29,30,31,32]. In addition, the trace elements Zr, Ti, V, Er and Sc added to the Al–Si–Cu–Mg cast aluminum alloys can not only refine grains, but also precipitate dispersed fine Al_3_M (M is Zr, Ti, V, Er or Sc) phase during aging heat treatment, and these precipitated phases have good precipitation strengthening effect [57].

**Figure 9:**
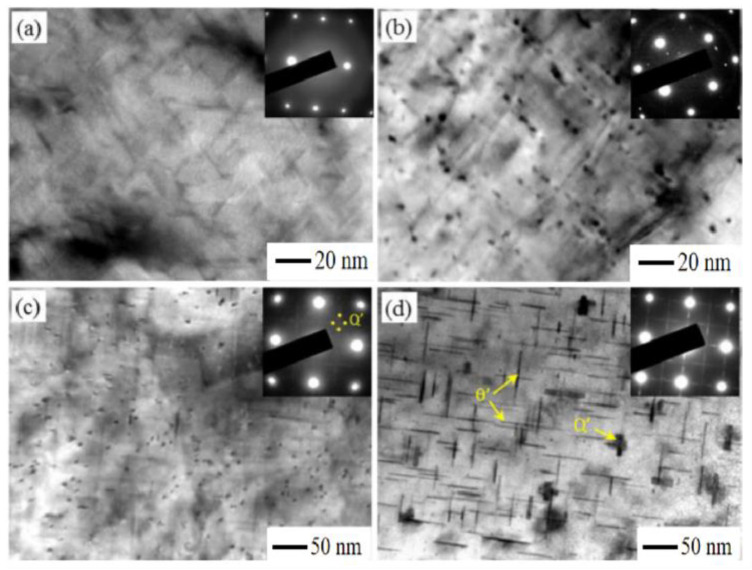
Bright-filed TEM image with corresponding SAED pattern of aged alloy [93]: (**a**) the precursor of the β’’ phase; (**b**) β’ phase; (**c**) Q’ phase; (**d**) Q’ and θ’ phase. TEM—Transmission Electron Microscopy; SAED—Selected Area Electron Diffraction.

Researchers have conducted a lot of research on the aging hardening of Al–Si–Cu–Mg cast aluminum alloys under different aging temperature and time. The regulation of aging hardening effect of Al–Si–Cu–Mg cast aluminum alloys is related to the type, density, size and distribution of precipitated phase in aging sequence [93,94,95,96]. For Al–Si–Mg cast aluminum alloys, the aging precipitation sequence is SSSS → atomic cluster → GP Zones → β″ → β’, U1, U2, B’ → β/Si [97]. Zhang et al. [98] found that β″ has a major strengthening effect on the alloy. For Al–Si–Cu cast aluminum alloys, the ageing precipitation sequence is SSSS → atomic cluster → GPI → GPII (θ’’) → θ’ → θ, including θ″ and θ’ are the main precipitation strengthening phase [99]. For Al–Si–Cu–Mg cast aluminum alloys, the aging precipitation sequence becomes very complex due to the increase of the types of aging precipitation phases. In addition to the above two precipitated sequences, the following aging sequences may occur: SSSS → atomic cluster → GP Zones → β″, L, QP, QC → β’,Q’ → Q. The types and volume fraction of the aged precipitated strengthening phase in Al–Si–Cu–Mg cast aluminum alloys are not only related to the heat treatment process, but also depend on the Cu content and Cu/Mg content ratio. Mørtsell et al. [100] studied the precipitation behavior of cast aluminum alloy A356 with different Cu contents and found that the precipitation strengthening phase of the A356 cast aluminum alloy has only the needle-shaped β″ phase under peak aging conditions; when 0.5 wt.% Cu is added to the alloy, L and Q’ appear in addition to the β″ phase; however, when 1 wt.% Cu is added to the alloy, there is only a small amount of β″ phase in the alloy, and the main precipitation strengthening phases are the L and Q’ phases. Zheng et al. [101] studied the precipitation strengthening phase of Al–Si–Cu–Mg cast aluminum alloys (Figure 9) and found that the alloy tends to form the precursor of the β″ phase when the low Cu (1.04 wt.% Cu) and Cu/Mg ratio is 2; when the Cu content is 1.06 wt.% Cu and the Cu/Mg ratio is 1, the alloy tends to form β’ phase; when the high Cu (2.08 wt.% Cu) and Cu/Mg ratio is 4, the alloy tends to form the Q’ phase; however, when the Cu content in the alloy reaches 3.98 wt.% Cu and the Cu/Mg ratio is still 4, in addition to a small amount of Q’, the θ’ phase exists in the alloy.

## 5. Conclusions

Cast aluminum alloy is one of the candidates for lightweight materials for automobiles. In recent years, the application of lightweight Al–Si–Cu–Mg cast aluminum alloys in automobiles has gradually increased, but it is limited by problems such as strength, toughness, cost, and application environment. The strengthening mechanism of cast aluminum alloy mainly includes solid solution strengthening, fine grain strengthening, and second phase strengthening. Generally, these strengthening mechanisms are related to solid atoms, grain size, and the second phase, respectively. However, microalloying and heat treatment play an important role in the regulation of microstructure and mechanical properties. Therefore, in order to make the development of Al–Si–Cu–Mg cast aluminum alloys for automotive lightweight towards the direction of low-cost, high strength and toughness, researchers systematically comprehend the method of optimizing microalloying and heat treatment based on the strengthening mechanism of cast aluminum alloy, and should pay attention to the following main aspects:

The optimization of multi-component alloying elements includes main alloying elements and microalloying, among which the optimization of main alloying elements (such as Si, Cu and Mg) are mainly related to solution strengthening and precipitation strengthening, and its research has been relatively complete. Optimizing the content of alloy elements, especially exploring the relationship between microalloying and strengthening mechanism is an important research direction to improve the mechanical properties at room temperature and high temperature. The regulation of microalloying on the microstructure of Al–Si–Cu–Mg cast aluminum alloys is mainly achieved by modifying eutectic Si, refining grain and forming new precipitation strengthening phase. Although microalloying has made some progress in the regulation of microstructure and mechanical properties, some high-efficient microelements are limited in their application due to high cost. For example, the rare earth element Sc has a good refining effect on eutectic Si and grain size, but the high cost of Sc impedes its wide use. Therefore, it is a future research trend to seek for low-cost and efficient trace elements to replace Sc or combine with Sc.

In the process of solution treatment of Al–Si–Cu–Mg cast aluminum alloys, the Si particles undergo crushing, spheroidizing and coarsening. However, the dissolution and homogenization of primary phase (such as Mg_2_Si phase, Al_2_Cu phase and Al_5_Cu_2_Mg_8_Si_6_ phase) are relatively complex. The dissolution of Mg_2_Si phase is very rapid, roughly between 15–50 min. The dissolution of Al_2_Cu phase is more difficult than that of Mg_2_Si phase, and the dissolution characteristic of Al_2_Cu phase is related to the morphology, in which the blocky Al_2_Cu phase is more difficult to dissolve than that of eutectic morphology Al_2_Cu phase. However, the dissolution characteristic of Al_5_Cu_2_Mg_8_Si_6_ phase is related to alloy composition and solution treatment parameters. The high Mg content causes the Al_5_Cu_2_Mg_8_Si_6_ phase to precipitate again during the solution treatment. In addition, the higher solution temperature is more beneficial to the dissolution of Al_5_Cu_2_Mg_8_Si_6_ phase than the lower temperature, but the higher solution temperature may lead to the overburning of Al_2_Cu phase. Therefore, the study on the dissolution kinetics mechanism of Mg_2_Si phase, Al_2_Cu phase and Al_5_Cu_2_Mg_8_Si_6_ phase in the solution treatment has theoretical guidance for the formulation of Al–Si–Cu–Mg alloy solution treatment, especially the two-step solution treatment.

The types and volume fraction of the aged precipitated strengthening phase (β″, Q’ and θ’ phase) in Al–Si–Cu–Mg cast aluminum alloys are not only related to the heat treatment process, but also depend on the Cu content and Cu/Mg content ratio. With the increase of Cu content, the strengthening phase gradually evolves from β″ phase to Q’ and θ’ phase. Exploring the mechanism of solid solution and dispersion strengthening in the heat treatment process of Al–Si–Cu–Mg cast aluminum alloys, especially the adjustment of aging precipitation sequence through alloy element design and heat treatment process optimization is an important means to improve the strength and toughness of the alloy.

## Data Availability

Not applicable.
